# Technical versatility of pedicled latissimus dorsi-based flaps for refractory spinal CSF leaks

**DOI:** 10.1016/j.jpra.2026.10.002

**Published:** 2026-10-22

**Authors:** Murtaja Satea, José Maria Zepeda Torres, Alaa Ibrahim Mohammed Jorani, Ahmed Mohammed Khaffaf, Jonathan Mokhtar, Elio Nader, Khaled Fares AlAli, Haïzam Oubari, Alexandre G. Lellouch

**Affiliations:** a Division of Plastic and Reconstructive Surgery, Cedars-Sinai Medical Center, Los Angeles, CA 90048, USA; b Department of Surgery, College of Medicine, University of Warith Al-Anbiyaa, Karbala 56001, Iraq; c Plastic Surgery Department, Unidad Médica de Alta Especialidad (UMAE) de Traumatología, Ortopedia y Rehabilitación “Dr. Victorio de la Fuente Narváez”. Instituto Mexicano del Seguro Social, México City, México; d Department of Plastic Surgery, Imam Hussein Medical City, Karbala 56001, Iraq; e College of Medicine, Mohammed Bin Rashid University of Medicine and Health Sciences, College of Medicine, Dubai Health, Dubai, United Arab Emirates; f Poznan University of Medical Sciences, Faculty of Medicine, UI Fredry 10, Poznan 61701, Poland; g Department of Neurosurgery, Zayed Military Hospital, Abu Dhabi, United Arab Emirates; h Division of Plastic and Reconstructive Surgery, Massachusetts General Hospital, Harvard Medical School, Boston, MA, USA; i Department of Plastic and Reconstructive Surgery, Croix-Rousse Hospital, University Hospital of Lyon, Lyon, France

**Keywords:** Latissimus dorsi flap, Pedicled flap, CSF leak, Spinal reconstruction, Flap surgery

## Abstract

**Background:**

Persistent cerebrospinal fluid (CSF) leakage following spinal surgery or congenital spinal reconstruction can result in pseudomeningocele, meningitis, wound breakdown, and exposed instrumentation. Management is particularly challenging after multiple operations or in the presence of poor-quality local tissue. When primary dural repair and conventional wound-management strategies fail, vascularized tissue transfer may be required to reinforce the dura, obliterate dead space, and promote durable healing.

**Methods:**

We report three patients with refractory spinal CSF leakage treated with pedicled latissimus dorsi-based flaps after failure of conventional treatment. Different flap configurations were selected according to defect location and reconstructive requirements, including a pedicled musculocutaneous flap, an extended muscle flap with fascial extension, and bilateral muscle advancement flaps with fascial extension.

**Results:**

All flaps survived and all CSF leaks resolved. Wound healing was achieved without major flap-related complications. Minor complications included seroma and limited skin-edge necrosis. Long-term follow-up was available in two patients at 2 years, demonstrating durable soft-tissue coverage without recurrent CSF leakage.

**Conclusions:**

Pedicled latissimus dorsi-based flaps represent a versatile salvage option for selected refractory spinal CSF leaks when primary dural repair and local reconstructive options are insufficient. Their configuration can be adapted to provide vascularized dural reinforcement and dead-space obliteration across different spinal levels.

## Presentation of innovation

The contribution of this report is not the introduction of a new flap, as the latissimus dorsi is an established workhorse for posterior trunk reconstruction, but rather the demonstration of its technical versatility in the specific setting of persistent spinal CSF leakage after failure of conventional treatment. Depending on defect location and reconstructive requirements, the flap can provide vascularized muscle for dural reinforcement, dead-space obliteration, and a robust biological barrier in potentially contaminated fields, with a skin paddle when additional resurfacing is required. The three cases illustrate how this established flap can be adapted to defects at different spinal levels in patients with limited local reconstructive options.

The flap is procured on the thoracodorsal pedicle. Its arc of rotation permits superior transfer to cervical and upper thoracic defects, medial rotation for thoracic and upper lumbar wounds, and inferior advancement or transposition for selected lumbar and lumbosacral defects. Technical refinements, including extensive pedicle dissection to the subscapular origin, wide lateral chest wall release, and creation of a high submuscular tunnel above the scapula, allow the flap to reach high cervical levels. For lumbosacral defects, additional length can be obtained through division of the humeral insertion and release of posterior axillary-line fascial restrictions.

To achieve maximal cranial reach for high cervical defects, the thoracodorsal pedicle is dissected proximally to its origin in the subscapular system, and the latissimus is released widely from the chest wall to maximize excursion. Depending on the patient's anatomy and local tissue quality, either a high suprascapular submuscular tunnel or a wide subcutaneous tunnel can be created to allow the flap to pass superiorly without pedicle kinking. Careful division of the tendinous humeral insertion provides additional length and eliminates tethering during rotation. While the latissimus dorsi is already an established reconstructive workhorse at the thoracic level because of its proximity and reliable vascular supply, the principal value of our series lies in demonstrating how this same donor muscle can be adapted when defects occur at the more challenging cranial (cervical) and caudal (lumbar and lumbosacral) levels.

Caudal reconstruction requires particular attention to both flap reach and distal perfusion because the latissimus becomes progressively thinner and more aponeurotic distally, while mobilization can interrupt some of its secondary segmental vascular contributions. In our lumbosacral case, bilateral latissimus dorsi muscle transposition flaps with fascial extension were used to recruit vascularized muscle over the dural defect without division of the thoracodorsal pedicle or incorporation of a distal skin paddle. Distally based turnover latissimus flaps relying on the secondary segmental vascular supply represent an alternative technique described for selected lower spinal defects but were not the configuration used in this case.^1^ Intraoperative flap perfusion was assessed clinically after flap mobilization and again following transposition and inset. Assessment included muscle color, capillary bleeding from the muscle edges, and the absence of clinical evidence of venous congestion. ICG angiography was not routinely used. Particular attention was paid to ensuring that the muscle positioned over the dural repair remained clinically well perfused and that the thoracodorsal pedicle was free of tension, kinking, or compression.

The latissimus dorsi should not be regarded as a universal replacement for established regional flaps. More broadly, complex salvage reconstruction often requires staged and multidisciplinary management when infection, compromised tissue quality, or major underlying pathology is present.^2^ Rather, its value lies in providing a reliable salvage option when local paraspinous, trapezius, gluteal, or fasciocutaneous tissues are insufficient, previously operated, scarred, or unable to provide the vascularized bulk required for durable dural coverage and dead-space obliteration.

In our series, three patients with persistent CSF leaks at different spinal levels underwent reconstruction using different configurations of pedicled latissimus dorsi-based flaps after failure of standard measures. All flaps survived, and CSF leakage resolved without recurrence during available follow-up, illustrating the versatility of latissimus dorsi-based reconstruction across different spinal levels.

### Case 1 – Cervical region

A 35-year-old woman developed a persistent CSF leak following C3–C5 dermoid tumor resection with prosthesis exposure. Primary dural repair and lumbar drainage failed. A pedicled latissimus dorsi musculocutaneous flap was harvested on the thoracodorsal pedicle and advanced superiorly through a subcutaneous tunnel (not submuscular) to the posterior cervical region. The muscle was placed directly over the dural repair to obliterate dead space and reinforce closure, while the skin paddle provided tension-free resurfacing. A Jackson-Pratt (JP) drain was inserted in the sub-flap plane and extended to the subcutaneous plane to decrease dead space and reduce seroma formation. The flap survived fully, the CSF leak resolved, and a small seroma — managed by aspiration — remained the only minor issue (Fig. 1).

**Fig. 1:**
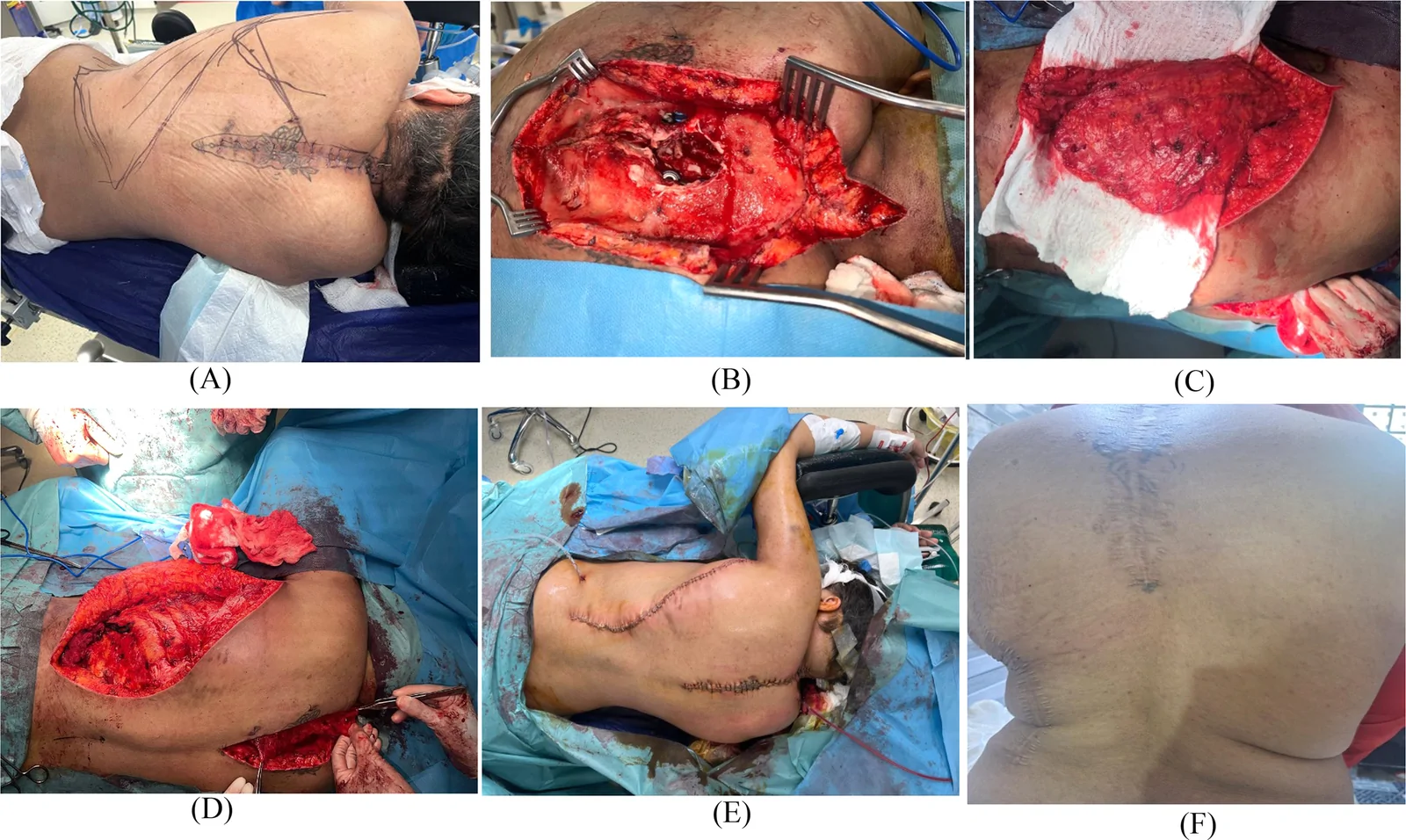
Pedicled latissimus dorsi musculocutaneous flap reconstruction in a 35-year-old woman with persistent CSF leakage following C3–C5 dermoid tumor resection with prosthesis exposure. **(A)** Preoperative markings outlining the planned latissimus dorsi flap and transfer pathway. **(B)** Intraoperative appearance of the posterior cervical defect after debridement and exposure of the underlying dural repair. **(C)** Elevated latissimus dorsi musculocutaneous flap demonstrating the vascularized muscle component. **(D)** Transfer of the flap toward the cervical defect through the subcutaneous tunnel. **(E)** Immediate postoperative appearance following flap inset and wound closure. **(F)** Long-term postoperative result demonstrating durable soft-tissue coverage and healed reconstruction without recurrent wound breakdown.

### Case 2 – Lumbar region

An 11-year-old boy presented with persistent CSF leakage after resection of a histologically confirmed L3–L5 intra-/extradural schwannoma. The wound demonstrated poor tissue quality with a deep midline defect. A pedicled latissimus dorsi muscle flap was harvested with a distal lumbosacral fascial extension to increase its effective reach and facilitate tension-free coverage of the defect. The flap was tunneled medially and inset using long-lasting absorbable PDS 3–0 sutures for secure fixation and dural reinforcement. A JP drain was placed in both the sub-flap and subcutaneous planes to reduce seroma risk. The flap survived completely, the CSF leak stopped, and the surgical site healed well without recurrence (Fig. 2).

**Fig. 2:**
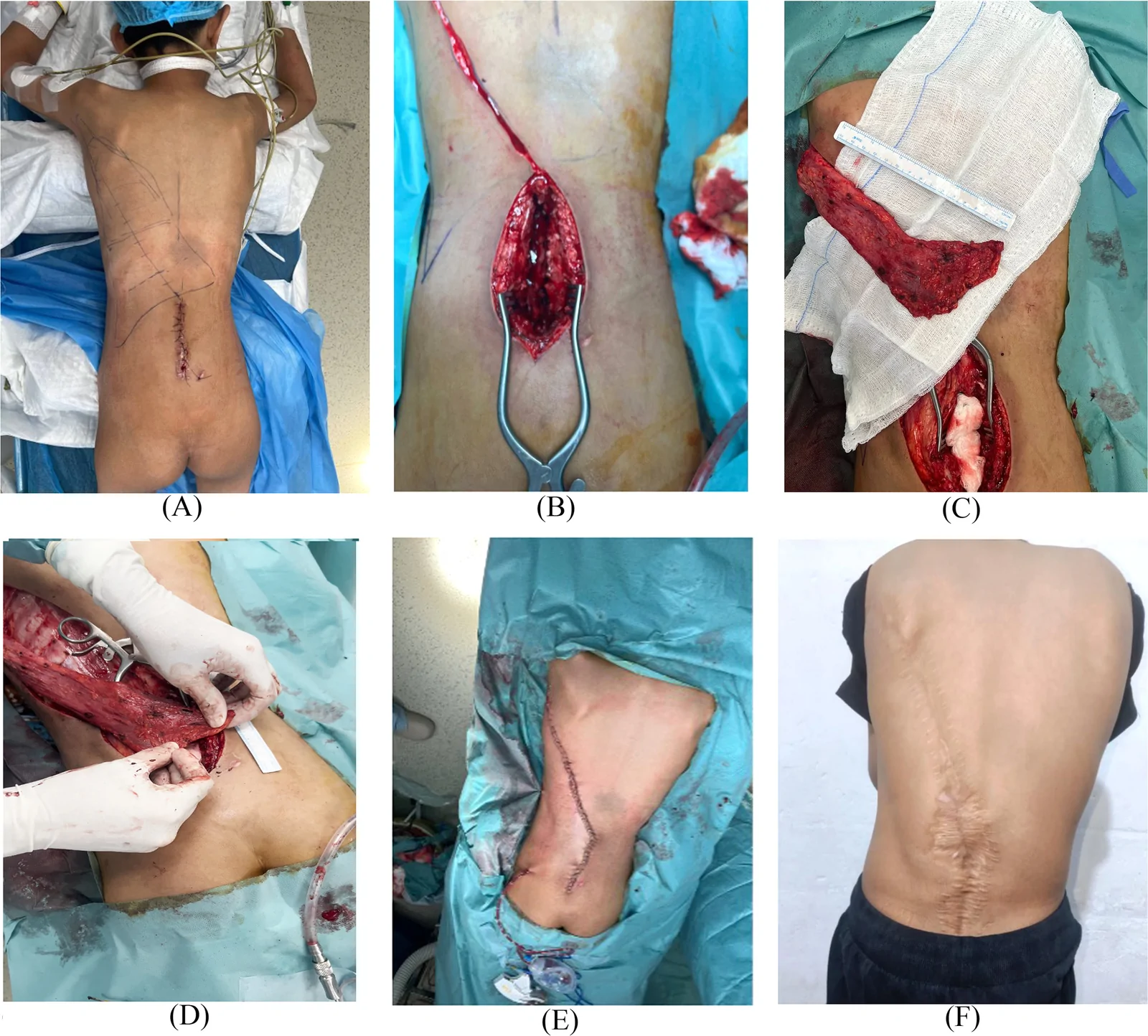
Pedicled latissimus dorsi muscle flap with distal fascial extension for reconstruction of a persistent lumbar CSF leak in an 11-year-old boy following resection of a schwannoma involving the L3–L5 levels. (A) Preoperative markings outlining the planned flap and lumbar defect. (B) Intraoperative exposure of the lumbar defect. (C) Elevated latissimus dorsi muscle flap demonstrating its distal fascial extension and available reach. (D) Medial transfer and inset of the flap toward the lumbar defect for dural reinforcement and dead-space obliteration. (E) Immediate postoperative appearance following wound closure. (F) Long-term postoperative result demonstrating durable soft-tissue coverage and healed reconstruction without recurrent wound breakdown.

### Case 3 – Lumbosacral region

A 6-month-old male infant with a persistent CSF leak following prior myelomeningocele closure and ventriculoperitoneal shunting underwent reconstruction using bilateral latissimus dorsi muscle advancement flaps. Both thoracodorsal pedicles were preserved. To increase caudal reach without sacrificing the thoracodorsal pedicles, the distal fascial extension of each latissimus dorsi muscle toward the iliac crest was preserved and incorporated into the advancement. The muscle bellies themselves were advanced medially and positioned directly over the compromised dura to provide vascularized coverage and obliterate the residual dead space, while the distal fascial extensions facilitated additional caudal excursion and tension-free inset. No skin paddle was incorporated. Donor-site closure was supported with JP drainage to minimize fluid accumulation. Healing was achieved with only a small area of approximately 1 cm of skin-edge necrosis, which resolved with conservative wound care. No further CSF leakage was observed during follow-up (Fig. 3). Postoperatively, no dedicated external compression was applied directly over the reconstructed spinal defect or flap. Closed-suction JP drainage was used to reduce dead space and fluid accumulation while avoiding direct pressure on the transferred tissue and vascular pedicle.

**Fig. 3:**
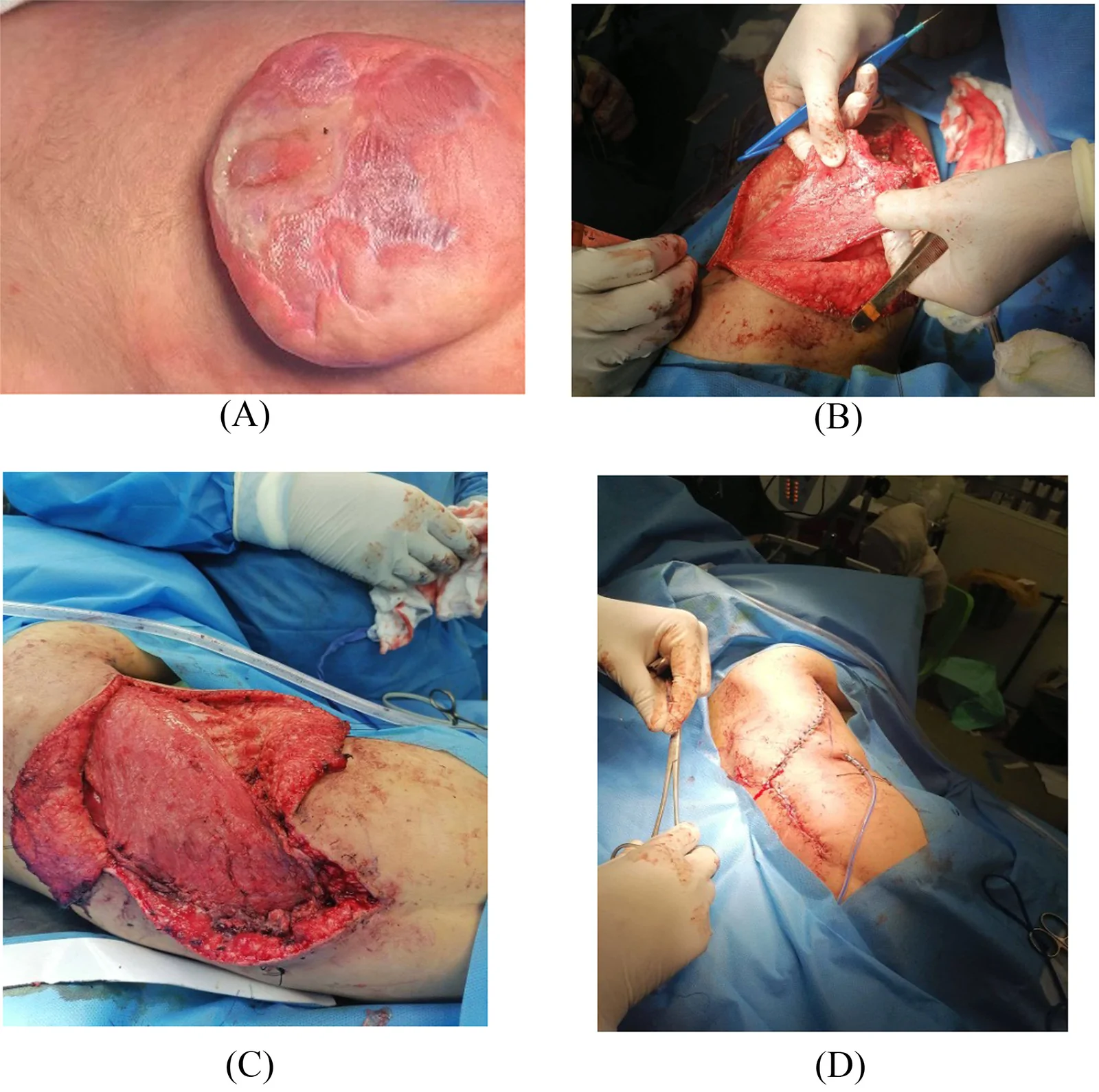
Bilateral pedicled latissimus dorsi muscle advancement flaps for reconstruction of a persistent lumbosacral CSF leak in a 6-month-old male infant following prior myelomeningocele closure and ventriculoperitoneal shunting. (A)Preoperative appearance of the lumbosacral defect. (B) Intraoperative elevation and mobilization of the latissimus dorsi muscle flap. (C) Bilateral medial advancement of the latissimus dorsi muscle flaps to provide vascularized coverage of the compromised dura and obliterate the residual dead space. (D) Immediate postoperative appearance following flap inset and wound closure.

Across all three cases, latissimus dorsi–based reconstruction provided vascularized tissue for dural reinforcement and dead-space obliteration, with successful resolution of persistent CSF leakage. Long-term clinical follow-up was available for two patients at 2 years and demonstrated durable soft-tissue coverage and maintained wound healing without recurrent CSF leakage **(Supplementary Fig. 1–3)**.

## Discussion

Persistent CSF leakage reflects not only a dural defect but also a failure of the surrounding soft tissue to support healing. Posterior trunk defects are particularly challenging due to limited local tissue, large dead space, and frequent prior surgery or radiation. In this setting, muscle and musculocutaneous flaps are preferred because they provide bulk and vascularity for durable coverage and dead-space obliteration.^3^^,^^4^ Local paraspinous and trapezius flaps remain important options for posterior cervical reconstruction and may be preferable for smaller, localized defects because of their proximity and limited donor-site dissection. The latissimus dorsi should therefore not be considered the default flap for cervical defects. Its advantages become more relevant when substantial vascularized bulk or a larger skin paddle is required, when the defect is extensive, or when local paraspinous or trapezius tissues have been compromised by previous surgery, radiation, infection, or recurrent wound breakdown. Flap selection should therefore be individualized according to defect size, spinal level, available regional tissue, previous surgery, and the volume of vascularized tissue required. At the opposite end of the spinal axis, gluteal-based flaps, including superior gluteal artery perforator flaps, are important alternatives for low lumbar and sacral reconstruction and offer the advantage of preserving muscle. They may be particularly attractive when the defect is predominantly cutaneous or fasciocutaneous and substantial muscle bulk is not required. In complex CSF leaks with a deep dead space or compromised dural coverage, however, a muscle-based reconstruction may be advantageous because it provides vascularized tissue that can be placed directly over the dural repair while simultaneously obliterating dead space. The choice between latissimus and gluteal-based reconstruction should therefore depend on defect location and depth, previous surgery, available regional tissue, and the need for muscle bulk rather than on a single-flap approach. Caudal use of the latissimus dorsi has important anatomical and vascular limitations. The dominant vascular pedicle is the thoracodorsal artery, while the more distal muscle also receives secondary segmental contributions from posterior intercostal, subcostal, and lumbar vessels. Watanabe et al. demonstrated distinct vascular territories within the latissimus dorsi, emphasizing the importance of these secondary vascular contributions when extended or distal portions of the muscle are mobilized.^5^ The muscle becomes progressively thinner and more aponeurotic distally, reducing the available muscle bulk for deep lumbosacral defects, while extensive mobilization may interrupt some of the secondary segmental vascular supply. Conversely, a distally based or turnover latissimus flap depends on preservation of these secondary segmental vessels, which may be disrupted by previous spinal surgery. When a distally based configuration is contemplated, the integrity of this blood supply should therefore be confirmed preoperatively or intraoperatively. In our lumbosacral case, we avoided reliance on a reverse or distally based configuration by preserving both thoracodorsal pedicles and advancing the bilateral latissimus dorsi muscle bellies, with their fascial extensions, over the dural defect.

Ramasastry et al. described principles for reconstructing posterior trunk defects, emphasizing the need for robust tissue to cover deep and complex wounds.^3^ Hochberg et al. demonstrated that muscle and musculocutaneous flaps effectively salvage exposed spinal fusion devices, underscoring the importance of vascularized flaps in complex spinal wound complications.^4^ These studies support the use of vascularized tissue in complex spinal wounds when primary closure is inadequate. Local paraspinous muscle flaps are frequently the simplest and most direct option because they use adjacent tissue, avoid a distant donor site, and can provide effective coverage with limited donor-site morbidity. The latissimus dorsi should not be viewed as a replacement for paraspinous reconstruction. Rather, we consider it particularly useful as a salvage option when local paraspinous tissues have been previously dissected, scarred, irradiated, infected, or are insufficient in volume to obliterate a large dead space. Its principal advantages in this setting are the availability of substantial vascularized muscle, a reliable vascular pedicle, and the option of incorporating a skin paddle when resurfacing is also required.

The latissimus dorsi is particularly useful in complex salvage reconstruction because it provides substantial vascularized tissue on a reliable pedicle and can be adapted to challenging defects when conventional local options are insufficient.^6^ Reverse and extended latissimus dorsi techniques have also been described for complex thoracolumbar and lumbosacral defects when local reconstructive options are exhausted.^7^ Systematic review data further support flap-based reconstruction for complex spinal wounds, demonstrating high rates of wound healing and successful salvage of spinal instrumentation in selected cases.^8^

In the pediatric and congenital context, McCraw and colleagues reported successful use of LDMFs for myelomeningocele closure, demonstrating that these flaps can provide durable coverage of exposed dura in large spinal defects.^9^ Jaworski et al. similarly reported successful back closure using an LDMF in a pediatric patient, reinforcing its reliability for posterior spinal defects in small children.^10^

Our three cases fit within this body of evidence: in each scenario—post-tumor resection, schwannoma resection, and myelomeningocele—pedicled latissimus dorsi-based reconstruction provided vascularized tissue for dural reinforcement and dead-space obliteration, resulting in durable CSF leak control. These cases illustrate the versatility of the latissimus dorsi as a salvage option across different spinal regions, but do not imply that it should replace established local or regional flaps. Its role is most relevant in selected high-risk wounds in which conventional dural repair has failed and local tissue is inadequate to provide durable vascularized coverage and dead-space obliteration. Flap selection should remain individualized according to defect location, previous surgery, tissue quality, and reconstructive requirements. This report has several limitations. First, it represents a small case series of three patients with heterogeneous etiologies, spinal levels, ages, and reconstructive requirements, precluding comparative assessment of the different flap configurations or of latissimus dorsi-based reconstruction against alternative regional flaps. Second, flap selection and operative technique were individualized for each defect and were therefore not standardized across cases. Third, objective intraoperative perfusion assessment with ICG angiography was not routinely performed, and flap viability was primarily assessed clinically. Finally, long-term follow-up was available in only two of the three patients. These limitations should be considered when interpreting the outcomes, and the present series is intended to illustrate technical options rather than establish superiority of one reconstructive approach.

## Ethical approval

Not required.

## Consent for publication

Written informed consent for publication of the patients’ clinical information and photographs was obtained from the adult patient and from the parents or legal guardians of the pediatric patients.

## Funding

No funding was received from any for-profit or not-for-profit organizations whose interests may be affected by the content of this work.

## Availability of data

Data is available upon reasonable request from the corresponding author.

## Declaration of competing interest

The authors declare no financial or commercial conflicts of interest related to the content of this manuscript.
